# An Overview of Antiviral Peptides and Rational Biodesign Considerations

**DOI:** 10.34133/2022/9898241

**Published:** 2022-05-17

**Authors:** Ying-Chiang J. Lee, Jaden D. Shirkey, Jongbeom Park, Karishma Bisht, Alexis J. Cowan

**Affiliations:** Department of Molecular Biology, Princeton University, Princeton, New Jersey 08544, USA

## Abstract

Viral diseases have contributed significantly to worldwide morbidity and mortality throughout history. Despite the existence of therapeutic treatments for many viral infections, antiviral resistance and the threat posed by novel viruses highlight the need for an increased number of effective therapeutics. In addition to small molecule drugs and biologics, antimicrobial peptides (AMPs) represent an emerging class of potential antiviral therapeutics. While AMPs have traditionally been regarded in the context of their antibacterial activities, many AMPs are now known to be antiviral. These antiviral peptides (AVPs) have been shown to target and perturb viral membrane envelopes and inhibit various stages of the viral life cycle, from preattachment inhibition through viral release from infected host cells. Rational design of AMPs has also proven effective in identifying highly active and specific peptides and can aid in the discovery of lead peptides with high therapeutic selectivity. In this review, we highlight AVPs with strong antiviral activity largely curated from a publicly available AMP database. We then compile the sequences present in our AVP database to generate structural predictions of generic AVP motifs. Finally, we cover the rational design approaches available for AVPs taking into account approaches currently used for the rational design of AMPs.

## 1. Introduction

From smallpox to dengue and influenza, viral agents and the diseases they cause have resulted in significant morbidity and mortality across the world and throughout the course of human history [1–3]. To counter these viruses, vaccines and antiviral therapeutics have been developed as prevention and treatment strategies, respectively. However, antimicrobial resistance (AMR) among viruses is of concern [4–7]. AMR, coupled with the heightened pandemic risk posed by certain viral pathogens as well as the threat posed by emerging viruses, underscores the need for an increased number of effective antiviral therapeutics as a complement and sometimes supplement for vaccines [8, 9].

AMR in the context of viral diseases is an important topic while garnering less attention than antibacterial resistance. Viral resistance against antivirals for HIV and HSV has been identified, and nearly all currently circulating influenza strains are resistant to M2 protein blockers [10–12]. In 2018, baloxavir marboxil (BXM), a new class of influenza drug, entered the clinic; however, by 2019, transmissible BXM-resistant influenza A strains were identified in the cities where BXM was initially approved [13, 14]. While viral strains resistant to vaccines are less of a concern, it should still be kept in mind especially in the face of breakthrough infections seen in the recent Omicron variant of SARS-CoV-2 [15, 16]. These examples highlight the ongoing and expanding threat of antiviral resistance.

In addition to antiviral resistance, emerging infectious diseases (EIDs) pose a significant risk to human health. Ebola Virus Disease (EVD) is a viral hemorrhagic fever and was discovered during two distinct outbreaks in 1976 of two different Ebola virus species [17]. Since then, multiple EVD outbreaks have occurred with the recent 2014 West African outbreak originating in Guinea and predominant transmission to several neighboring countries infecting more than 28,000 individuals and causing more than 11,000 deaths [18]. While EVD is notable with its high mortality rate, various coronaviruses have demonstrated elevated mortality rates or demonstrated pandemic potential. Severe acute respiratory syndrome (SARS) was first identified in southern China in late 2002 and had an estimated case fatality ratio of 11% [19]. Another novel coronavirus was identified in Saudi Arabia, subsequently named Middle Eastern Respiratory Syndrome (MERS), and found to be transmitted from camels and currently has a case fatality rate of 21% [20, 21]. In mid-December 2019, a cluster of cases of what is now designated severe acute respiratory syndrome coronavirus 2 (SARS-CoV-2) emerged in Wuhan, China. The viral disease SARS-CoV-2 causes COVID-19, which became the world’s first coronavirus pandemic within months and continues to spread [22].

The threat of increased antiviral resistance and the health risk of EIDs warrants further research into novel antiviral compounds. Occupying the space between small molecules and biologic drugs, peptides are a potential source for new, clinically relevant, antivirals. Antimicrobial peptides (AMPs) are a broad class of peptides which exhibit antimicrobial activity by perturbing pathogen membranes, regulating pathogen or host protein machineries, or both [23, 24]. The majority of known AMPs have been naturally isolated from the innate immune systems from a range of organisms, but an exciting area that warrants further research is the engineering of synthetic AMPs using rational design strategies. Most documented AMPs are cationic, range from 10 to 100 amino acids long, and commonly form amphipathic *α*-helical or disulfide-driven *β*-sheet conformations which drive their interactions with target proteins or membranes.

While most AMPs have typically been characterized in the context of their antibacterial activities, many AMPs are now known to be antiviral. Antiviral peptides (AVPs) have been shown to affect the viral replication cycle through several mechanisms: by perturbing the membranes of enveloped viruses; inhibiting cellular penetration or intracellular trafficking; restricting viral transcription and translation; and/or preventing budding of mature viral particles [25–27]. While AVPs are a prospective source of new antiviral therapeutics, some exhibit high cytotoxicity against human cells *in vitro*, making their clinical adoption difficult without further efforts to improve their selectivity through peptide engineering of lead candidates. An increasing number of AVPs are being rationally designed using structural and ligand-based design strategies [28–30]. For example, rationally designed, low cytotoxicity AVPs have been created to specifically bind to viral proteins such as protruding glycoproteins and spike proteins to prevent viral-host interactions, interfere with enveloped viral membranes, or inhibit viral proteases [31–33].

Thousands of natural and synthetic AMPs have been curated into online databases such as APD3, DRAMP 2.0, CAMP-R3, dbAMP, and ADAM [34–38]. However, only a small subset of entries in these databases document antiviral activity and few databases are exclusive to AVPs (AVPdb and HIPdb) [39, 40]. Total unique peptides in these databases can range from several thousand to over 29,000 peptides with the non-AVP focused databases only containing a small fraction (ranging from 1 to 7%) of AVPs, and often lack accessible antiviral activity values. Perhaps the most recognizable AMP database is APD3. APD3 contains over 3000 unique AMP entries that span six kingdoms of life with bioactivity against bacteria, viruses, fungi, and parasites [38]. Approximately 83% of the AMPs in APD3 are antibacterial while only about six percent are antiviral (including anti-HIV) as of the most recent version of the database. The relatively low proportion of documented AVPs could be because currently discovered AMPs are not effective against viruses in general or that most AMPs have only been tested for antibacterial activity. If the latter is true, researchers should be encouraged to broadly test AMPs that they discover or design against both bacteria and viruses and to thoroughly document all antiviral activity, including a lack of activity, in centralized databases. While the majority of AMP databases include variations of peptide prediction and design tools based on general peptide parameters, there is a need to develop AVPs that display high selectivity that may not be reflected in algorithms analyzing traditional peptide parameters. Specificity of AVPs can be better achieved through the rational design and screening of AVPs using *in silico* and *in vitro* approaches.

In this review, we consolidated AVPs cataloged in the antimicrobial peptide database APD3, including mutants and other AVPs found during literature searches of bioactivities for the APD3 AVPs. We examined a total of 280 unique AVPs among 371 total AVP entries with links to APD3-derived AMPs and identified experimentally validated antiviral activity values from primary sources for a majority of entries. We also documented cytotoxicity values from primary sources along with cell types used in experiments, if available. Peptide parameters such as length, charge, Bowman index, and hydrophobicity were also included. We highlight several AVPs with strong antiviral activity according to the viruses they target, with a focus on HIV, influenza A virus, as well as several emerging viruses. We then provide an analysis of the sequences in our database, visualizing predictions of conserved AVP motifs that serve as a basis for further AVP research. Finally, we survey approaches for rational design of AVPs, with a particular focus on AVP structure, function, design, and limitations.

## 2. Viral Pathogens and AVPS with Demonstrated Activity

### 2.1. Human Immunodeficiency Virus (HIV)

HIV is a single-stranded retrovirus that infects humans and can lead to the development of acquired immunodeficiency syndrome (AIDS) over time. The virus enters cells through the CD4 receptor primarily expressed by T cells, monocytes, macrophages, and dendritic cells. After the initial infection, HIV gradually reduces the number of CD4 cells in the body during the asymptomatic stage until the cell count reaches a critical threshold, upon which an individual is said to have AIDS [41, 42]. At this point, serious illnesses develop including opportunistic viral, bacterial, and fungal infections as well as certain types of cancer [43, 44]. Regardless of the extent of disease progression, antiretroviral therapy (ART) is the standard of care treatment for all individuals with HIV [45, 46]. ART is also prescribed for high risk individuals seeking out preexposure prophylaxis (PrEP) [47]. There are three common ART regimens available to patients, with each regimen including a combination of small molecule inhibitors. These include nucleoside analog reverse transcriptase inhibitors (NRTIs), integrase strand transfer inhibitors, and/or an HIV protease inhibitor. NRTIs prevent the virus from converting its RNA-based genome into DNA while integrase strand transfer inhibitors prevent the virus from inserting its genome into the host genome [48, 49]. Protease inhibitors block both the enzymatic activity and dimerization of the HIV protease [50]. These pharmacologic agents decrease viral load in the blood and help to prevent transmission but do not eradicate all viral reservoirs in the body. New antivirals may help add to the current treatments for HIV/AIDS, potentially acting synergistically in some cases.

During our indexing process, we identified a number of AVPs with sub-micromolar antiviral activity, some of which are seen in Table 1. Several mutants of the cationic polyphemusin II peptide originally isolated from horseshoe crabs show high anti-HIV activity. The 18 residue T22 mutant was found to have a median effective concentration (EC50) of 0.0026 *μ*M and a median toxic concentration (TC50) of 17.8 *μ*M in MT-4 cells [51]. Although the exact antiviral mechanism is yet to be determined, it is believed that T22 targets viral cell fusion or uncoating. Another polyphemusin II derivative designated T140 had an EC50 ranging from 0.0035 to 0.012 *μ*M and a CC50 ranging from 45 to 54 *μ*M [52]. A 21 residue long, negatively charted peptide from Streptomyces bacteria called siamycin I showed antiviral activity with a median effective dose (ED50) of 0.08 *μ*M and a TC50 of 150 in CEM-SS human T4 lymphoblastoid cells [53]. Siamycin I was determined to function as an AVP by reversibly inhibiting HIV-induced fusion. A group of circulin peptides from the Chassalia parviflora plant with net charges ranging from 0 to 2 and containing multiple disulfide bonds was found to also be highly active against HIV in vitro. Circulins A-F displayed EC50s between 0.04 and 0.26 *μ*M with circulins A and B having a cytotoxic IC50 of 0.5 *μ*M [54]. While the mechanism of anti-HIV activity displayed by circulins has not been determined, the location of positive charges in the peptides are thought to impact peptide-membrane interactions and result in the antiviral activity observed [55].

**Table 1:** Select AVPs with activity against different viruses. Multiple AVPs display high antiviral activity against various viral pathogens. Antiviral and cytotoxic activity values listed here and in our database were either directly stated or inferred from dose-response curves in the sources. Abbreviations: MeV: measles virus; pseudo-EBOV: recombinant vesicular stomatitis virus expressing EBOV-GP.

AVP	Virus	Antiviral activity (*μ*M)	Cytotoxic activity (*μ*M)	Reference
Siamycin I	HIV	0.08 (ED50)	150 (TC50)	[53]
Polyphemusin II (T22)	HIV	0.0026 (EC50)	17.8 (CC50)	[51]
Tachyplesin II (T11)	HIV	0.151 (EC50)	16.86 (CC50)	[101]
Tachyplesin II (T15)	HIV	0.0375 (EC50)	22.85 (CC50)	[101]
P9	IAV(H1N1)	0.36 (IC50)	113.88 (TC50)	[81]
Mucroporin M1	IAV (H5N1)	1.03 (EC50)	41.03 (CC50)	[83]
Urumin	IAV (H1N1)	3.8 (IC50)	2,450 (TD50)	[86]
HNP-1	IAV	0.58 (IC50)	N/A	[76]
P9	MERS-CoV	1.5 (IC50)	113.88 (TC50)	[81]
P9R	SARS-CoV-2	0.26 (IC50)	87.9 (CC50)	[82]
Latarcin 1	DENV	8.3 (EC50)	52.51 (CC50)	[102]
An1a	ZIKV	2 (IC50)	>40 (TC50)	[98]
GI-20d	Pseudo-EBOV	0.99 (IC50)	18.8 (TC50)	[100]
17BIPHE2	Pseudo-EOV	0.71 (IC50)	13.2 (TC50)	[100]
Mucroporin M1	MeV	3.52 (EC50)	30.31 (CC50)	[83]
Temporin B	HSV-1	1.8 (EC50)	65 (CC50)	[103]
Mundticin KS	HSV-2	3.5 (EC50)	3,497 (CC50)	[104]
Epinecidin-1	FMDV	0.26 (EC50)	8.35 (CC50)	[105]

The AVPs mentioned here mostly interact with viral fusion and membrane interactions. When used in combination with current small molecule HIV antivirals, they could present as attractive combinatorial therapies while minimizing toxicity. A larger library of validated HIV-targeting AVPs may help in the optimization and design of future HIV antiviral therapeutics.

### 2.2. Influenza A Virus (IAV)

Influenza A virus (IAV) is a single-stranded, segmented RNA virus that affects the pulmonary system. In addition to seasonal epidemics, IAV strains can occasionally cause global pandemics. Seasonal IAVs particularly affect young children and the elderly and are responsible for 3-5 million cases of severe illness and 650 thousand deaths every year worldwide [56, 57]. Moreover, the pandemic and highly pathogenic avian IAVs, such as the 1918 pandemic virus and the viruses of the H5N1 strain, respectively are known to cause more severe disease in healthy adults than seasonal IAVs, often resulting in mortality in humans [58–60]. Additionally, the presence of short aberrant replication products of IAV like the miniviral RNAs can further complicate disease progression [61]. The associated costs with these infections lead to an annual economic loss of hundreds of billions of dollars in the US alone [62]. Currently, there are two classes of antiviral drugs known to be effective against IAV infection, the neuraminidase inhibitors and the M2 ion channel inhibitor [63–65]. However, widespread and near universal resistance has been reported for these against the majority of circulating IAV subtypes [12, 66–73]. Additionally, in 2018 and 2019, baloxavir marboxil (BXM), a new class of influenza drug, which is known to target the polymerase of IAV, showed reduced effectiveness and emergent resistance against several variants of IAV [13, 14, 74].

With the emergence of drug-resistant IAV, the unique and promising antiviral activity of some of the documented AMPs makes them a preferred choice over the already available antiviral agents to combat IAV infection. In this section, we will discuss how some of the known AVPs reported in the antimicrobial peptide database APD3 act as antiviral agents against IAV [38]. The two most important cationic host-defense peptides in mammals are defensins and cathelicidin. Defensins are a family of small antimicrobial peptides comprising *α*, *β*, and *θ* subfamilies. Defensins are important for host defense by modulating the innate immune response. Additionally, they demonstrate both antibacterial and antiviral properties. The most common antiviral mode of action, in vitro, is the capacity of these AMPs to neutralize and aggregate various strains of IAV and increase their uptake by neutrophils [75, 76]. The median inhibitory concentration (IC50) value for different subtypes of *α* defensins (HNP1–4, HD5, HD6) ranges from 0.58 *μ*M to 1.43 *μ*M, while for human *β* defensin 1 (hBD-1) and human *β* defensin 2 (hBD-2) it is reported to be <8.39 *μ*M and <3.46 *μ*M respectively, in A549 cells. Similarly, another important human defense peptide is cathelicidin LL-37 which has shown potent antiviral activity against IAV by reducing viral replication as well as virus-associated inflammation in infected mice. Also, LL-37 demonstrated a 60% survival rate compared to the saline or scrambled peptide control in in vivo mouse infection model [77, 78].

Other than the AVPs of human origin, there are also peptides obtained from nonhuman species showing potent antiviral activity against IAV. For example, mouse *β*-defensins like mBD1 and mBD3 are known to prevent IAV infection by directly blocking virus attachment and cell entry. Additionally, the mBD1-mBD3 recombinant protein can inhibit influenza A virus replication both in vitro and in vivo effectively [79, 80]. Another short peptide derived from mouse *β*-defensin-4 is P9, which shows broad-spectrum antiviral effects against IAV. P9 is mainly composed of basic amino acids and it acts by inhibiting virus-host endosomal acidification, thereby preventing viral escape from endosomes. The antiviral activity for P9 against influenza virus H1N1 was evaluated in MDCK cells and the IC50 and TC50 were reported to be 0.36 *μ*M, and 113.88 *μ*M, respectively [81]. Recently, another modified version of P9 peptide, called P9R was reported with increased net positive charge via arginine substitutions. P9R was found to inhibit viral replication by binding to IAV and preventing acidification in endosomes. P9R also showed a 70% protection in in vivo mice infection model as compared to the PBS-treated mice. Additionally, this peptide prevented the emergence of a resistant mutant virus even after 40-virus passages, suggesting a very low possibility for P9R to generate a drug-resistant virus [82].

In addition to the above-mentioned AVPs, there are also some nonmammalian sources of peptides effective against IAV. For instance, mucroporin M1, a cationic host defense peptide from the venom of scorpion Lychas mucronatus has an EC50 of 1.03 *μ*M and a median cytotoxic concentration of viable cells (CC50) of 41.03 *μ*M against the H5N1 strain of IAV, in MDCK cells [83]. Although the exact mechanism of action is unknown, the direct interaction of mucroporin with the virus envelope is speculated to cause a decrease in the infectivity of the virus. Likewise, another peptide from the whole plant of Viola yedoensis, cycloviolacin VY1, displayed anti-influenza A H1N1 virus activity in in vitro assay. The IC50 value for this peptide was reported to be 0.704 *μ*M [84]. Similarly, alloferin 1 and 2, two peptides isolated from the blood of an experimentally infected insect, showed potent antiviral activity against IAV in a mice lethal pulmonary infection model by utilizing their immunomodulatory properties [85]. Urumin represents another unique host defense peptide obtained from the skin of a south Indian frog. It specifically targets the cell surface protein, hemagglutinin, causing viral disruption and is effective against drug-resistant H1 influenza viruses. With an IC50 value of 3.8 *μ*M and a TD50 of 2,450 *μ*M, the antiviral effect of urumin was specific only towards IAV as opposed to some other RNA viruses like Ebola or HCV [86]

IAV infections, especially those associated with highly pathogenic viral strains, pose a significant threat to the world economy and our healthcare systems. With the current antiviral drugs being susceptible to drug resistance and the limited vaccine efficacy, the development of novel, alternative therapeutics is of utmost importance. AVPs have demonstrated antiviral activity against several IAV subtypes and could therefore represent one of the potential classes of new antiviral agents against IAV infection.

### 2.3. Emerging Viruses

In addition to HIV and IAV, we identified and cataloged a number of AVPs that are mentioned in the literature on viral pathogens that cause emerging infectious diseases (EIDs). Due to the often sporadic nature of EID emergence, potential for high mortality rate, and evidence of rapid transmission in some cases, effective antivirals are needed. Coronaviruses that cause SARS, MERS, and COVID-19 belong to a large family of enveloped, positive-sense, single-stranded RNA viruses. While the initial SARS and MERS outbreaks were contained within months or displayed low transmissibility, COVID-19 quickly became a pandemic in early 2020 and continues to spread worldwide with the highly transmissible and vaccine-eluding Omicron variant consisting of the majority of new infections [87, 88]. In addition to coronaviruses, other pathogens such as the Ebola, Zika, Dengue, and Chikungunya viruses present as public health threats. Ebola virus is an enveloped, negative-sense, single-stranded RNA virus that causes viral hemorrhagic fever with an average case fatality rate of 50% [89, 90]. Zika virus and Dengue viruses are both enveloped, positive-sense, single stranded viruses belonging to the Flaviviridae family. Zika was previously thought to be a mild disease but epidemiological studies and the widespread infection in the Americas now show that Zika virus can cause neurological effects in adults and is a cause for microcephaly in children whose mothers are infected with the virus during pregnancy [91, 92]. Dengue virus infection causes a mild to moderate disease for most people with symptoms but a second infection with another of the four Dengue serotypes increases the chances of life threatening dengue hemorrhagic fever [93]. Chikungunya virus is not normally lethal but can result in debilitating and severe joint pain and arthritis [94, 95]. The potential for severe morbidity and mortality coupled with ease of transmission and expected acceleration of EID emergence highlights the need for effective treatments [96].

AVPs are an attractive option that can substitute or complement small molecule antivirals. All of the EIDs mentioned here are enveloped and thus present a primary target for AVPs in addition to viral protein binding and inhibition. Table 1 includes some of the AVPs detailed here. P9, a 30 residue peptide derived from the mouse beta defensin 4, was determined to have IC50s of 1.5 *μ*M against both SARS-CoV and MERS-CoV and an IC50 of 0.719 *μ*M against SARS-CoV-2 with TC50 of 113.88 *μ*M in MDCK cells [81, 82]. P9 was found to bind to the spike glycoprotein S2 of MERS-CoV and possibly inhibit viral RNA release. P9R, a modified version of P9 with a more positive net charge that we have mentioned earlier in this review led to a drop in the IC50 for SARS-CoV-2 to 0.264 *μ*M and a CC50 of 87.9 *μ*M in MDCK cells [82]. Similar to P9, P9R was found to prevent viral binding as well as inhibit virus-host endosomal acidification seen in viral replication for pH-dependent viruses. Figainin 2, a 28 residue peptide isolated from the skin secretions of the Chaco tree frog has an EC50 of 17.9 *μ*M against chikungunya virus [97]. While the exact mechanism is undetermined, Figainin 2 is thought to act similarly to other AVPs from frogs through viral membrane disruption. A 36 residue AVP, An1a, from the venom of the spider Alopecosa nagpag displayed activity against both Zika virus and Dengue virus with IC50s of around 2 *μ*M and 10 *μ*M, respectively, with cell viability values at over 40 *μ*M in human umbilical vein endothelial cells [98]. An1a was shown to inhibit the NS2B-NS3 protease of Zika and Dengue viruses. The 76 residue scorpion peptide Smp76 also displayed activity against Zika and Dengue viruses with IC50s of around 6 *μ*M [99]. Instead of being membrane active, Smp76 was found to upregulate IFN-*β* expression and type-I IFN response demonstrating an immunomodulatory AVP mechanism. In a pseudo-Ebola virus (pseudo-EBOV) infection model in HeLa cells, the cathelicidin LL-37 was found to have an IC50 of 4.03 *μ*M and a TC50 of 25.1 *μ*M [100]. Composed of 20 D-amino acids, the engineered LL-37 AVP GI-20d blocked pseudo-EBOV cell entry by inhibiting cathepsin B, a cysteine protease required for EBV cell entry, and was found to have an IC50 of 0.99 *μ*M and TC50 of 18.8 *μ*M in HeLa cells [100].

## 3. AVP Structure, Function, Design, and Limitations

Most AMPs that exist in publicly available databases originate from naturally derived sources [38]. However, to facilitate the discovery of novel, improved, and functionally diverse AVPs, protein design strategies can be used to engineer peptides which specifically interact with viral or host biomolecules of interest [106]. Recent advancements in structural biology and computational chemistry have provided a rapidly growing number of experimentally validated or predicted protein structures, facilitating the rational design of novel AVPs by allowing researchers to visualize structures of target proteins, designed peptides, and their interactions [107]. AVPs have been engineered entirely de novo, but computational techniques (like template-based design and virtual screening) and combinatorial methods (using directed evolution techniques) are often employed in series to further optimize lead peptides [29, 108–111]. Designing an AVP for therapeutic use begins with establishing a desired mechanism of action, thus dictating the biological target. Structural and computational data can then be used to rationally design candidate peptides which bind to and modulate the target. Finally, the peptide(s) must be produced, purified, assayed for antiviral efficacy, and eventually formulated for patient delivery.

### 3.1. Structural Analysis of Diverse AVPs

Although AVPs can be highly diverse in their mechanisms, intriguingly, certain structural characteristics are shared by many AVPs. Analysis of the 280 AVP sequences in our assembled database suggests that most AVPs are under 100 residues long, with an average length of 31 amino acids. AVPs commonly have strong net positive charges at physiological pH but are also largely amphipathic, with the average AVP sequence in our database displaying a +4 charge while being composed of nearly half hydrophobic residues. The AVPs in our database are likely biased to be membrane-disrupting peptides, as evidenced by their average Boman index of 1.24; additionally, 100 of the 280 unique peptides are predicted to generate an *α*-helical structure, per the APD3 database [38].

**Figure 1:**
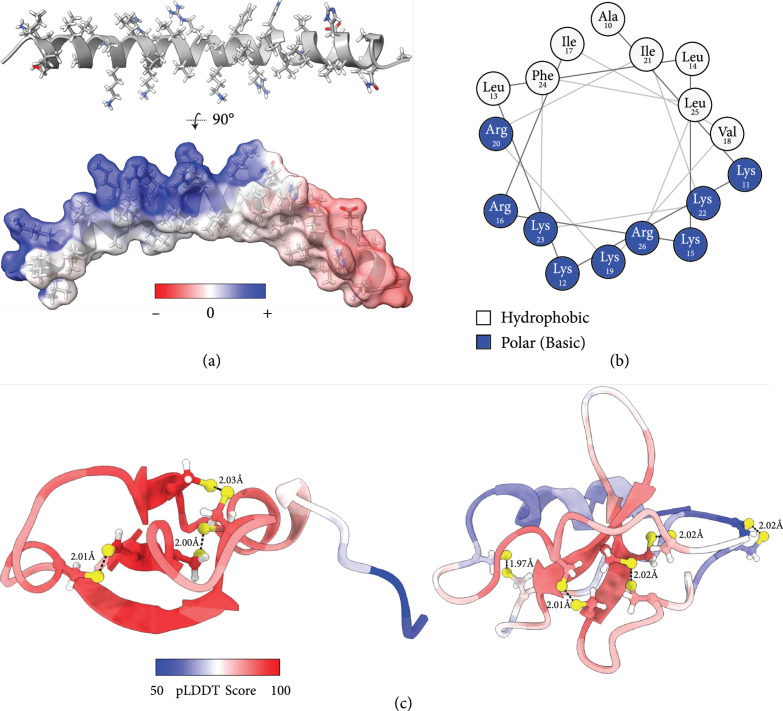
Visualization of common AVP motifs via structural prediction of consensus sequences. Three peptide consensus sequences were isolated from the 280 AVPs in our database (via Clustal Omega multiple sequence alignment), and their structures were predicted *in silico* using AlphaFold 2.1.1 and visualized with UCSF ChimeraX. (a) An *α*-helical AVP model derived from CS1 (top) with its strongly amphipathic surface shown (bottom). Side chains are colored by heteroatoms; the surface is colored by electrostatics calculated from the Adaptive Poisson-Boltzmann Solver [120]. (b) A NetWheel cross section characterizing residues 10-26 of the helix shown in (a). (c) Two cysteine-rich *β*-sheet AVP models derived from CS2 (left) or CS3 (right). Cysteine side chains are shown and colored by heteroatom, and predicted disulfide bonds are drawn as dashed lines with lengths labeled in Angstroms. Residues are colored by normalized pLDDT confidence scores, where values of <70 should not be interpreted, values of 70-90 indicate confident backbone predictions, and scores>90 suggest highly accurate side chain placements.

By performing a Clustal Omega multiple sequence alignment (MSA) on the AVP sequences we have collected, three consensus sequences were identified (Table 2). AlphaFold 2.1.1 was used to computationally predict the three-dimensional structures of these representative consensus sequences, enabling the visualization of generic motifs which commonly result in antiviral activity. The strongest consensus sequence (Consensus Sequence 1; CS1) in Table 2 gives rise to an entirely *α*-helical motif which displays a striking amphipathic conformation (Figure 1(a)), where one helical face is exclusively composed of hydrophobic residues, while the opposite face is highly charged from Lys and Arg residues (Figure 1(b)). Peptides with these structural qualities can have detergent-like characteristics and may act directly as virucides or bactericides by disrupting and solubilizing pathogen membranes [112]. This class of peptides has also been shown to function by inhibiting viral entry, intracellular trafficking, or viral exit, through various mechanisms which are described in detail below [82, 108, 110, 113].

**Table 2:** Antiviral peptide consensus sequences identified from our AVP database. Three conserved sequence motifs were identified from the 280 sequences composing our AVP database. Structural characteristics were determined via AlphaFold 2.1.1 predictions, seen in Figure 1. “# AVPs” column refers to the number of unique AVP sequences which contributed to the calculated consensus sequence. Peptide weight and theoretical isoelectric points were calculated from ExPASy ProtParam. Abbreviations: CS: consensus sequence ; pI: isoelectric point.

ID	AVP consensus sequences	Characteristics	Peptide size	pI	# AVPs	Related AVPs
CS1	GLLSKLGSLAKKLLKRIVKRIKKFLRNHVVPVIAEHLVGANA	Amphipathic *α*-helix	42 residues, 4630 Da	11.8	100	BMAPs, caerins, GF-17, LL-37, mucoporins, temporins, uperins
CS2	GVHGGGFGGGGGGFGGNNPNRCLTNGGICWRRRGPCPTKGRQIGNCGHAKVRCCKIR	Glycine-rich motif; cysteine-rich *β*-sheets (3 sheets)	57 residues, 5810 Da	10.9	31	P9R, polyphemusin II, procambarin, urumin
CS3	RDVALRERRGGQCRGGGPCGESCFRGCCRGICYRGGCSCRYQVRPRWKVCYRNGSCPIIIGRC	Cysteine-rich *β*-sheets (2 sheets)	63 residues, 7050 Da	9.6	123	Circulins, cycloviolacins, protegrins, retryocyclins, tachyplesins, siamycins

The other two consensus sequences (CS2 and CS3) outlined in Table 2 share 34% sequence similarity (as calculated by the EMBOSS Water Pairwise Sequence Alignment tool), and gave rise to structural predictions with highly similar qualities. Both models are dominated by *β*-sheet secondary structures (Figure 1(c)), and are enriched in cysteine residues, which compose 10.5% and 17.5% of their respective sequences (compared to 0% of CS1). These cysteine residues are paired in the structural predictions, such that each residue has a partner at an appropriate distance to facilitate disulfide bond formation [114]. These disulfide bonds help to stabilize the *β*-sheet conformations, and have also been shown to provide resistance against protease degradation [115]. A glycine-rich motif is also present along the N-terminus of CS2, and appears unstructured and highly flexible in the AlphaFold prediction (Figure 1(c), left model, blue termini); however, it should be noted that this region is entirely derived from the sequences of three AVPs (alloferon, procambarin, and shepherin II), and is not present on the other 28 AVPs which contributed to the cysteine-rich beta-sheet structures seen in CS2. Although not pictured, the surface projections of these models resemble disc-like densities (due to disulfide-driven peptide cyclization), and contain large patches of both hydrophobic and cationic residues.

Out of the 280 sequences analyzed, the vast majority aligned with one of the two main structural classes shown in Figure 1—amphipathic *α*-helices or cysteine-rich *β*-sheets. Although these two classes of peptides may look quite different at first glance, specific sequence changes can allow either class to perform similar functions—for example, strongly amphipathic *β*-sheets can function to solubilize membranes, while certain *α*-helices may function by binding viral protein machinery. Ultimately, the chemical and physical properties of an AVP are dictated by its specific amino acid sequence, thus defining its three dimensional shape, binding partners, and its resulting mechanism of action [111, 116–119].

### 3.2. AVP Mechanisms of Action

Membrane disruption is an attractive AVP mechanism against enveloped viruses because these peptides are likely to be broad-action, directly virucidal, and target the most accessible region of a virion. However, because the membranes of enveloped viruses originate from their host, the greatest challenge in developing these drugs is achieving selective disruption of viral membranes [121]. Peptides which directly perturb or solubilize membranes require strong amphipathic chemical characteristics. For example, a 12-residue lytic peptide designed entirely from arginine and valine residues (RVVRVVRRVVRR) was de novo engineered to form an ideal amphipathic helix with strongly defined polar and nonpolar faces, in a similar manner to the consensus sequence visualized in Figures 1(a) and 1(b) [122]. Similarly, amphipathic cysteine-rich *β*-sheets, such as naturally derived protegrins and defensins, have also been shown to perturb microbe membranes [123]. Hydrophobic interactions stabilize an AVP against the pathogen membrane, with tryptophan residues playing a notable role due to the large surface area of indole rings [122]. Most AMPs, and 83% of the AVPs in our database, are biased to be positively charged along their polar faces. This may be due to the net negative charges present on the outer membranes of gram-negative bacteria (originating from the carboxylate and phosphate moieties on the underlying lipopolysaccharide (LPS) layer), as well as on enveloped virus glycoproteins [124, 125]. Correspondingly, it has been observed that increasing the ratio of arginines in a peptide is correlated with increased AVP activity, which is likely related to its high pKa and positively charged state at physiological pH [82, 111].

Once associated with a target membrane, amphipathic AVPs may self-oligomerize, potentially inserting themselves into membranes to form hydrophilically lined pores. Peptides may do this through different mechanisms—notably, through the barrel stave and toroidal pore models—but the common result is membrane deformation and disruption, impeding the ability of the viral membrane to fuse with its host cell [126–128]. Above a critical concentration, these amphipathic, helical peptides may directly solubilize pathogen membranes by inducing micelle formation, stabilized by both hydrophobic interactions (between nonpolar AVP side chains and envelope lipid tails), and electrostatic interactions (between cationic AVP residues and anionic phospholipid heads) [129, 130]. As mentioned, solubilization of enveloped virus membranes is perhaps the most direct way to generate a virucidal peptide drug.

Amphipathic, highly basic helical peptides have also been described to inhibit the intracellular trafficking of diverse pH-dependent viruses including enveloped viruses such as SARS-CoV-2 or influenza viruses as well as nonenveloped viruses like rhinovirus [81]. By binding to viral surfaces, these peptides travel with the virion into an endocytic vesicle where they function to resist the host process of endosomal acidification, ultimately delaying or preventing the activation of pH-triggered viral fusogen machinery which is required for viral escape into the cytoplasm. Though their mechanism is not clearly defined, it is possible that these AVPs hinder acidification via their electrostatic contributions to the endosomal lumen, resulting in resistance to the influx of hydrogen ions (driven by H+ ATPase pumps). Alternatively, these AVPs may partially act as a buffering agent by sequestering the incoming luminal protons - the amino acid histidine, with its pKa of 6.0, is theoretically the most effective amino acid for this task. Finally, these AVPs might regulate endosomal acidification by directly interacting with the host machinery which drives this process; such an example is the 62 residue, cationic peptide derived designed from the N-terminus of the human transferrin receptor, which is believed to enhance endosomal acidification by binding G_i_-protein coupled receptors upstream of the proton pump in its activation cascade [131].

A distinct mechanism of viral neutralization is the prevention of viral entry into a host cell. One way this can be achieved is by designing AVPs which function as attachment-inhibitors, blocking the docking of viral glycoproteins onto host cell receptors. For example, several peptides derived from the human ACE2 receptor have been shown to antagonize SARS-CoV-2 entry by competitively binding to the Receptor Binding Domain (RBD) of the viral spike protein, ultimately inhibiting the virion’s ability to infect cells [108, 132, 133]. Similarly, a 20-residue peptide was found to broadly diminish type A Influenza viruses from entering mammalian MDCK cells by binding a pocket on the viral glycoprotein hemagglutinin, reducing the virion’s affinity for host sialic acid receptors [134]. The West Nile virus has also been targeted in this manner [135]. Inversely, AVPs may be designed as host-cell receptor blockers, thus sterically antagonizing virion docking. A hexapeptide (YKYRYL) encoding a fragment of the SARS-CoV spike protein RBD has been shown to inhibit the in vitro infection of Vero E6 cells by blocking the human ACE2 receptor [136]. Furthermore, the FDA-approved small molecule drugs aplaviroc, maraviroc, vicriviroc, and cenicriviroc inhibit HIV entry through this method by binding the human CCR5 protein [137].

One of the most recent classes of antiviral peptides are those which block envelope virus entry by directly inhibiting viral fusogen machinery. The first FDA approved AVP in this class was enfuvirtide, a 36 residue peptide that mimics portions of the HIV-1 protein gp41 - a membrane-embedded, viral fusogen which relies on oligomerization to function [138]. After gp41 docks to host receptor CD4 (and coreceptor CCR5), gp41 undergoes dramatic conformational rearrangements, inserting itself into the host plasma membrane, thus driving membrane fusion and viral entry [139]. AVPs which mimic the fusogen’s *α*-helical domains that mediate oligomerization can competitively bind to fusogen monomers during these rearrangement events, preventing proper quaternary assembly and subsequent formation of a fusion pore [140]. Similar to AVPs which target membrane disruption, a benefit of fusion inhibitors as antiviral candidates is that they target the virus before cytoplasmic entry [138]. Although different enveloped viruses (such as HIV-1 and influenza) utilize structurally conserved machineries to fuse with their host [141], these machineries lack appreciable signal homology and evolve over time, leading to AVPs which are unlikely to be broad-action and may be prone to resistance development but can exhibit high specificity with reduced off-target effects [138]. For example, fusogen-mimetic, lipidated AVPs that respectively target HIV-1, influenza A, hepatitis B and D, and the measles virus have been described, with IC50 values as low as the single-digit picomolar range [142].

Many AVPs function by inhibiting viral enzymes required for viral transcription, translation, or posttranslational processing, ultimately disrupting the viral replication process. Similar to fusion inhibitors, AVPs which target viral enzymes have the benefit of potentially being highly specific, and are excellent candidates for rational design. Unlike fusion inhibitors, enzymatic inhibitors can be designed to target any virus (e.g., nonenveloped viruses). However, these peptides can be particularly difficult to formulate for patient delivery, as the peptide must be delivered to pathogenic enzymes residing within the cytosol of infected host cells. Nevertheless, many inhibitory antiretroviral drugs have undergone FDA approval, generally targeting proteases, reverse transcriptases, or integrases.

Viruses often synthesize their proteins as polyprotein chains, which are subsequently cleaved via viral or cellular proteases; thus, inhibition of these proteases is a viable AVP strategy [143, 144]. Over three decades ago, peptide derivatives (containing hydroxyethylamine functional groups) were rationally designed to competitively target HIV-1 and HIV-2 proteases. These AVPs inhibited viral proteases in the nanomolar range, yet did not influence human proteases at concentrations as high as 10 *μ*M [145]. Today, there are nine clinically approved HIV protease inhibitors, and seven of them (with the exception of Tipranavir and Darunavir) are peptide-derived drugs which bind the protease active site by mimicking enzymatic transition state intermediates [146, 147].

Other enzymatic inhibitors may target viral transcriptases or integrases, impeding the process of viral expression and replication [148]. Although all of the thirteen FDA-approved drugs which inhibit HIV’s reverse transcriptase are synthetic small molecules (including eight nucleoside/nucleotide derivatives (NRTIs) and five structurally unique molecules (non-NRTIs, NNRTIs)), peptides have also been demonstrated to inhibit reverse transcriptases by mimicking portions of their surfaces, competitively disrupting their ability to homo-dimerize [149, 150]; this is conceptually similar to the mechanisms of fusogen inhibitors described above. Related AVPs have been designed to target integrases, ribonucleotide reductases, or even host ribosomal subunits, thus demonstrating the diversity of candidate target proteins which can be chosen for antiviral development [151–153].

Finally, it is worth mentioning nonpeptide modifications which may complement and enhance novel AVP development. AVP lipidation can facilitate and direct interactions with target membranes, either to induce membrane permeability and disruption [154], or to localize antifusogenic peptides to their site of action [155]. Noncanonical amino acids or other synthetic moieties can be used to modulate peptide structures, target specificity, and half-life [147, 156–158]. Furthermore, small molecule drugs, oligonucleotides, or engineered antibody constructs such as Single Chain Variable Fragment fusions or nanobodies may be conjugated to AVPs to promote diverse antiviral functions and modulate the peptide’s therapeutic effects, localization, or bioavailability [112, 159, 160].

### 3.3. Computational and Combinatorial Design Strategies

**Figure 2:**
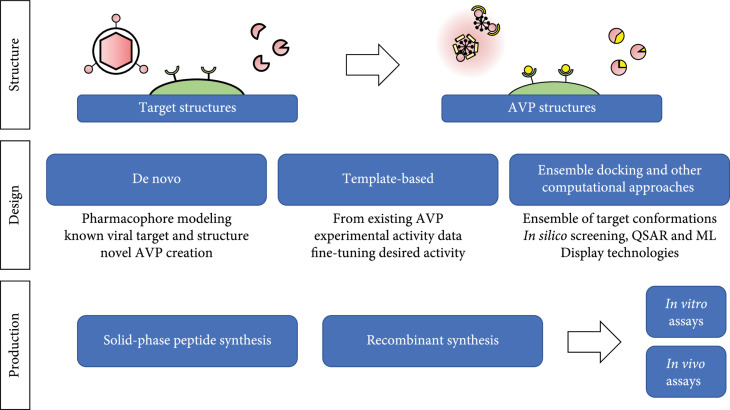
Engineering and production of rationally designed AVPs. Designing AVPs requires knowledge of the potential viral target structure that the AVP will bind or interact with. Next, structure-based rational design of AVPs can be performed via de novo, template-based, and/or ensemble docking approaches, in conjunction with combinatorial techniques. Finally, the designed AVPs must be produced through recombinant or solid-phase synthesis, purified, and subsequently assayed for efficacy.

An indispensable tool in the rational design of a novel AVP is computationally driven structural visualization. Historically, a major bottleneck in the design of novel ligands has been a lack of available structures against target-proteins-of-interest [161, 162]. Previously, homology modeling has been used to circumvent this problem (where orthologous target structures are used in place of the desired structure), but recent advances in cryo-electron microscopy and computational chemistry have made structural information more accessible than ever [163]. Despite the 51-year-old history of the Protein Data Bank (PDB), nearly a quarter of its experimentally determined structures have been deposited within the last 4 years (https://www.rcsb.org/stats). Furthermore, major breakthroughs in artificial intelligence systems have led some to claim the protein-structure prediction problem “solved” with the release of DeepMind’s AlphaFold at CASP14 in December 2020 [164], as this program can accurately predict the structures of certain protein monomers. Computational predictions have already been generated for 21 model proteomes, and DeepMind aims to generate more than 130 million structural models by the end of 2022 [165]. Although diverse techniques may be used to facilitate the development of novel AVPs, most strategies begin with a structural analysis of the desired target. Here we will cover several strategies used to guide novel AVP design, including de novo design, template-based design, computational screening approaches, and combinatorial experimental techniques (Figure 2).

The most direct way to engineer an AVP from a target structure is through de novo design: the process of determining a peptide sequence “from scratch”, using pharmacophore modeling to draft an AVP’s sequence one residue at a time [166]. This process, perhaps the pinnacle of rational drug design, theoretically enables the synthesis of truly novel AVPs against novel targets. Generally speaking, when targeting a protein such as a key viral receptor, fusogen, or enzyme, one must identify suitable binding sites to design an AVP against. These sites may include known active sites, allosteric sites, or sites that mediate protein-protein interactions. On a protein’s surface, exposed protrusions, deep clefts or pockets, or areas with pronounced chemical characteristics may all be leverageable features when designing a cognate AVP [167, 168]. When considering the synthesis of membrane-disrupting AVPs, membrane modeling and molecular dynamics simulations may be used; others have relied on predicted peptide structures to generate ideal amphiphiles [122]. De novo design has been successfully used to generate an incredible array of functional peptides, including broad-action membrane-disturbing AMPs, attachment-inhibitors which block SARS-CoV-2 entry, *α*-helical peptides which target Class I enveloped virus fusogens, as well as others [29, 110, 122, 169–171].

De novo and ab initio peptide designs have also been achieved through structurally agnostic design strategies. Instead of relying on atomic models of pathogen targets to reverse engineer an AVP, databases of AVP sequences (with their respective efficacies) have been analyzed and filtered to identify AVP characteristics which correlate with a desired mechanism and pathogen specificity. These characteristics, such as amino acid composition, peptide length, percent hydrophobicity, total charge, and disulfide content, can then be used to guide the design of novel sequences. This approach enabled the design of small, highly hydrophobic, and minimally cationic AVPs which specifically target MRSA via membrane-perturbation [172]; additionally, de novo sequences containing only Gly, Leu, and either Lys or Arg were engineered to specifically and respectively target either *E. coli* or HIV-1 [173].

A related structure-based strategy relies on designing an AVP by using a previously generated template sequence. Such a template might be a first-generation, de novo designed AVP; alternatively, naturally occurring AVP sequences may be used as references in designing novel peptides [174]. To rationally design an optimized AVP based on a template sequence, it is highly desirable to have experimentally validated structural data which visualizes interactions between the template AVP and the target of interest, enabling researchers to predict how educated sequence changes may modulate target binding. In the absence of structural data, ligand-based design strategies can be employed, relying on the knowledge of other molecules that tightly bind the target to guide the design of a related but novel peptide [175]. Utilizing template-based approaches expedites the design process relative to de novo synthesis, and can result in improved drug candidates which exhibit increased target affinity relative to the template used, potentially resulting in improved therapeutic indices and efficacy.

A third structure-based design strategy useful for AVP development is the computational approach of fast molecular ensemble docking. This process relies on the formation of virtual collections, or ensembles, of a target protein’s conformational states (determined through molecular dynamics simulations or structural biology techniques such as NMR), which can be combined with similar ensembles of candidate peptide sequences and conformations. These ensembles are computationally intermixed and screened to predict protein-peptide interactions. By scoring these interactions using various parameters (e.g., the number and type of interactions, solvent-accessible surface areas, knowledge-based statistical potentials, or other AI-derived parameters), lead peptide candidates can be identified. This approach aims to account for the dynamics of a protein’s structure, which may otherwise be ignored when exclusively using experimentally derived or predicted structural snapshots. As peptides are generally larger in size than small-molecule drugs, and display enormous degrees of freedom, this process can quickly become computationally overwhelming. One method of reducing the scope of such simulations is to impose conformational limitations through the use of rotamer libraries; others have developed algorithms, such as MedusaDock, which comodel peptide and protein conformations simultaneously [176–178].

Additional computational and statistical approaches have been instrumental in the development of antiviral or antimicrobial drugs [178]. First developed in 1962 [179], quantitative structure–activity relationship (QSAR) modeling is a regression method utilized by medicinal chemists which functions to correlate and predict how chemical characteristics dictate their resulting behavior [180, 181]. This approach has guided the rational design of AVPs which inhibit the human ACE2 receptor, as well as AMPs which broadly target gram-negative bacteria [182, 183]. Diverse machine learning algorithms have also been employed to facilitate peptide design, by predicting peptide structures, identifying ‘hotspot residues’ which strongly dictate peptide-protein interactions, or generally predicting the likelihood that a peptide sequence may demonstrate AVP qualities through evolutionarily or database comparison [184–189]. For additional reviews which expand on these computational approaches, please see the following references [190–194].

While traditional peptide rational design approaches can be powerful and yield highly efficacious AVPs, combinatorial approaches are often employed in series to facilitate drug discovery [195]. Combinatorial techniques introduce randomness to broaden a candidate peptide’s sequence diversity, and combined with high-throughput screening, can be an exceptionally powerful way to optimize candidate AVPs. Perhaps the most common combinatorial technique employed is phage display. Phage display is a directed evolution technique, relying on phage libraries made up of viruses which respectively express a single candidate AVP on their capsid surface [196]. Random mutagenesis is used to mutate this library, leading to the expression of diverse (but related) peptides. These peptides are then screened against or selected for their ability to bind to their target, allowing the identification and subsequent isolation of successful candidates. These candidates may be subjected to additional mutagenesis, leading to iteratively improved drugs. This technique goes hand-in-hand with rational design approaches, as designed peptides may dictate the initial phage library preceding mutagenesis; alternatively, rational design may be following phage display to further improve the most successful candidates [197, 198]. Phage display is of particular interest in designing AVPs, as peptide expression on viral surfaces is well tolerated, can enable the screening of billions of candidate sequences, and is adaptable to multiple stages within the drug design workflow [198].

Other combinatorial approaches include related display methods, such bacterial display, yeast display, mRNA display, or ribosome display. The former is most similar to canonical phage display but differs in that the engineered proteins of interest are generally expressed as OmpA fusions localized to the outer membrane of E. coli, instead of on a viral capsid. This technique has the benefit of enabling flow-assisted cell sorting (FACS) as an efficient screening and isolation platform [199]. Yeast display is a closely related technique which places the recombinant peptides on the Aga2p protein which is present on the exterior of the yeast cell wall. Yeast display serves as a superior platform for the evolution of larger, eukaryotic proteins, which may require eukaryotic chaperones and posttranslational modifications for proper folding. The mRNA display approach is of particular interest in the development of novel AVPs as it better allows for incorporation of noncanonical amino acids or peptide derivatives due to its in vitro nature [200]. Furthermore, while bacterial and phage display methods are capable of generating libraries of ~109 candidate peptide sequences, in vitro approaches like mRNA and ribosomal display are not limited by host transfection efficiencies, and are able to facilitate enormous libraries as vast as ≥1015 candidate sequences [201]. Briefly, as the name suggests, mRNA display uses a peptide’s mRNA as its display platform by covalently linking a candidate peptide to its mRNA precursor through a puromycin linkage, present on the 3${}^{\prime }$ terminus of the mRNA. This mRNA-peptide fusion is assayed for its ability to bind its desired protein target, and binders are isolated (e.g., by affinity chromatography). Error-prone PCR is performed to generate cDNA libraries based on efficacious peptide sequences, which can then be enzymatically transcribed and modified to give new mRNA libraries with a 3${}^{\prime }$ puromycin linkage, allowing the process to repeat. Lastly, ribosomal display uses a highly similar approach to mRNA display, enabling a mRNA-peptide linkage not with puromycin, but through the removal of the stop codon on the mRNA, ultimately resulting in the formation of a ribosome-mRNA-candidate peptide complex, which can then be assayed, isolated, and amplified [202].

### 3.4. AVP Challenges and Limitations

While we have covered essential aspects of rational AVP design, there are limitations to peptide-based therapeutics that may hinder the research, development, and use of AVPs. Only about 70 peptide drugs have entered the US market since the discovery of insulin in 1983 [156, 203], partly because of the challenges of peptide design, inherent peptide limitations, and viral resistance. Until recent years, rational peptide design was hindered by the absence of structural information for viral protein targets, especially structures containing a bound AVP ligand that could facilitate template-based design. Peptide design, synthesis, and purification is an expensive and laborious workflow which may fail entirely to generate sequences which effectively bind the desired target.

Even if a candidate AVP is shown to binds its target using biophysical assays such as ELISAs, surface-plasmon resonance spectroscopy, biolayer interferometry, or isothermal calorimetry, a candidate AVP may fail to neutralize its target virus in cells or in the body. For example, physiological salt concentrations have been shown to impact AMP activity [204, 205]. Some strategies are available that might mitigate the effect of salts, such as peptide cyclization, net charge reduction, and strategic amino acid substitutions [206, 207]. Another common problem peptide drugs face is susceptibility to proteolytic cleavage [140]. However, synthetic peptides, such as those with noncanonical amino acids, synthetic attachments (such as N-terminal polyamides), or cysteine-rich peptides with a split *α*/*β* structure are less prone to proteolytic degradation than strictly *α*-helical or unstructured peptides [200, 208]. Although a candidate AVP may display picomolar affinity to its target and resist proteolysis and harsh pH environments, it may be observed to promiscuously bind proteins in the body or cause nonspecific membrane damage that leads to undesired clinical side effects. This is documented with AMPs such as magainin and mastoparan, but peptide engineering efforts can be applied to design noncytotoxic peptides [209, 210]. Modifications to hydrophobicity and amphipathicity of peptides along with strategic amino acid substitutions have all been shown to enhance cell selectivity towards microbial targets over eukaryotic and red blood cells [210–213], and rational design strategies may be implemented on existing AVPs to further optimize their therapeutic effects.

Finally, drug formulation and delivery mechanisms are another set of challenges that must be considered when developing novel AVPs for therapeutic use, as both impact peptide stability, bioavailability, and therapeutic safety. For example, peptide drugs may be unstable in certain temperatures, pH, or ionic environments, leading to the formation of decomposed byproducts or aggregates [214]. Drug delivery mechanisms are important to consider, as they influence peptide doses, dosing frequencies, pharmacokinetic profiles, locality of side effects, patient compliance, and interpatient variation [215]. These mechanisms may be dictated by the viral target—for some viruses like influenza, inhalation is the most direct way to deliver therapeutic peptides to the viral targets [27], while for most other viral infections examined in this review, a fast, systemic intravenous (IV) delivery may be warranted. If different delivery platforms are possible, their respective pros and cons must be considered: oral delivery is favorable by patients but requires large doses and risks peptide inactivation or destruction in the gut; inhalable drugs may avoid gastrointestinal inactivation, but drug formulation is particularly challenging; intranasal delivery may provide prophylactic benefits but requires peptides with long half-lives; IV administration is the least accessible delivery for the patient but provides superior bioavailability and delivery speed. Thus, aspects of AVP design, efficacy, production, stability, and delivery all contribute to the challenges of developing novel AVPs.

## 4. Conclusion

Antiviral therapeutics are desperately needed. The rising issues of antiviral resistance combined with the potential for emerging viral diseases that threaten global health such as Ebola, Zika, and SARS-CoV2 all highlight the need for a robust and sustained research of antiviral therapeutics. AVPs present an attractive therapeutic space for further research and may be used solely or in combination with traditional small molecule antivirals. Many AVPs are mined and cataloged into extensive databases, often with a lack of readily accessible experimental values for antiviral and cytotoxic activity that could guide work in developing AVP-based therapeutics. Here, we surveyed all AVPs listed in APD3 including any derivatives we came across during the literature search and cataloged available experimental antiviral and cytotoxic values. This served as the database we used to select and describe select AVPs that demonstrated a high degree of antiviral activity for HIV, HSV, IAV, and EIDs. We then cover rational design of AMPs in general with a focus on strategies for rational design of AVPs based on drug targets.

While there are a handful of AVPs that seem to hold potential as antivirals, many of which are described in this review, most are not known to be in clinical development. This could be due to a number of reasons, including the challenges that peptide therapeutics face such as stability and possibility of toxicity in in vivo models. Peptides are susceptible to degradation, from acids to proteases that are secreted in high amounts in the gastrointestinal tract, thus reducing bioavailability and making oral delivery of peptide therapeutics including AVPs especially difficult. Another hurdle to consider includes the possibility of immunogenicity resulting from peptide therapeutics. While immunomodulation by AMPs is well documented and often acts to amplify the antimicrobial effects, immunogenicity, where an immune system mounts a targeted response against the administered AVPs, can reduce the effectiveness of the AVPs themselves and cause an immune response to naturally occurring host AMPs such as defensins and cathelicidins if the therapeutic AVPs are derived from host AMP sequences.

Despite these challenges, the collection of highly active AVPs documented in this review may serve as the backbone for future AVP development utilizing peptide engineering and innovative delivery techniques. While these peptides display potent activity and, in some cases, favorable selectivity indices, a design-centric approach may offer increased on-target effects and decreased cellular toxicity. AVPs specifically designed to target viral entry by inhibiting spike or cleavage and entry-essential proteins or viral replication complexes could provide a high degree of specificity. In addition, advances in the field of in silico protein folding and protein interaction prediction programs could help aid in peptide-based drug design. The future of AVPs will most likely be design-focused, drawing inspiration from nature, either in the form of existing AVPs or from viral targets and can help AVPs leave an increased footprint in antiviral therapeutics.

## Data Availability

The AVP database we have assembled is available upon request. Please direct inquiries to the corresponding author.
